# American Medical Association

**Published:** 1891-06

**Authors:** 


BUFFALO MEDICAL AND SURGICAL JOURNAL

A MONTHLY REVIEW OF MEDICINE AND SURGERY.

*EDITORS:*

THOMAS LOTHROP, M. D. - - WM. WARREN POTTER, M. D.

All communications, whether of a literary or business character, should be addressed to the editors : 284 Franklin Street, Buffalo, N. Y.

Vol. XXX. JUNE, 1891. No. 11.

### Editorial.

### AMERICAN MEDICAL ASSOCIATION.

The forty-second annual meeting of the American Medical Association, held in Washington, May 5, 6, 7, and 8, 1891, was one of its best. Probably it would not be stating the matter too strongly to say that in some respects it was the very best meeting the Association ever held.

Two conditions contributed to make the meeting particularly notable, namely: First, its President, Dr. W. T. Briggs, of Nashville, who performed the duties of his office in a manner most acceptable, displayed great tact and parliamentary skill in his disposition of the business, and particularly in dealing with those estimable gentlemen who annually seek to weary the house with their display of windy nothingness, and who generally succeed in embarrassing the Chair with their persistency in seeking his eye and ear. Woe betide such men when Dr. Briggs swings the gavel. The President, in this instance, combined the rare qualifications of professional ability, ripe judgment and parliamentary knowledge.

The second felicitous fact that contributed to success was the cool weather which prevailed during the entire session. Usually the first week in May is hot in Washington, and ill suited to the performance of intellectual work, but this time overcoats and fires were in demand.

The attendance was not as large as when the meeting was held in Washington in 1884, but the number was ample for the performance of good work, and the sections were well attended.

The officers elected for the ensuing year were: President, Dr.

Henry O. Marcy, of Boston; 1st Vice-President, Dr. Willis P. King, of Missouri; 2d Vice-President, Dr. Henry Palmer, of Wisconsin; 3d Vice-President, Dr. W. E. B. Davis, of Alabama; 4th Vice-President, Dr. W. E. Taylor, of California; Treasurer, Dr. Richard J. Dunglison, of Pennsylvania; Secretary, Dr. Wm. B. Atkinson, of Pennsylvania; Librarian, Dr. Geo. W. Webster, of Illinois; Trustees, Drs. W. W. Dawson, of Ohio, W. W. Potter, of New York, and J. H. Rauch, of Illinois. The nominating committee was fortunate in the selection of its chairman, Dr. W. H. Wathen, of Louisville, whose rare skill and judgment, as well as intimate knowledge of the affairs of the Association, derived from long experience, guided it safely through its multifarious duties without serious conflict or misunderstanding. The Committee agreed upon Hot Springs as the next place of meeting, but the house, on motion of Dr. H. O. Walker, of Michigan, struck out Hot Springs and inserted Detroit. So the place of meeting in 1892 will be Detroit, Michigan, where it will convene on the first Tuesday in June.

Dr. H. O. Walker was chosen chairman of the Committee of Arrangements, which is a guaranty that the Association will be courteously and kindly cared for during its forty-third annual session. We note but one error named in the list of nominees, and that was in choosing Dr. Didama, of New York, to the place on the Judicial Council. As Dr. Didama was not present at the meeting, he is ineligible under the rules.

The absorbing questions which first confronted the Association was that pertaining to the place of publication of the *Journal*, and who should be placed at its editorial head. The Trustees wisely decided that there should be no change in the *Journal's* home, and so the general session was relieved of a perplexing discussion, which would have inevitably followed if the subject had been thrown into the house. The Trustees were unable to come to a conclusion in Washington as to the editor, hence adjourned to meet in Chicago, May 13, 1891. At this meeting the Board of Trustees elected Dr. J. C. Culberston, of Cincinnati, to be editor, and to manage the business of the *Journal* as well. Dr. Culbertson has been for a number of years the editor and owner of the Cincinnati *Lancet-Clinic*, hence brings experience, as well as ripened judgment and editorial reputation, to his new field. We have no doubt that the *Journal* will continue to prosper under his skilful guidance. Taken all in all, the forty-second annual meeting of the American Medical Association may be regarded as a pronounced success, one long to be remembered for the scientific work which it accomplished, as well as the wisdom that governed in its deliberations over some of the assuredly important and trying affairs that per tain to such a representative body of American physicians.

NOTES OF THE MEETING.

The election of Dr. Henry O. Marcy, of Boston, to the presidency was a fitting recognition of the services of one of its most faithful members and indefatigable workers. Dr. Marcy always attends the meetings and contributes largely to their scientific interest each year. We hardly see how the Association could have done otherwise than to choose him as its first officer.

Dr. Charles A. L. Reed, of Cincinnati, introduced his resolution providing for a continental congress of physicians and surgeons, which was passed without opposition. A committee of one from each State and Territory was chosen to carry out the details of the organization, and this committee subsequently most properly elected Dr. Reed its *Chairman;* Dr. Carhart, of Texas, *Secretary;* and Dr. Love, of Missouri, *Treasurer*. We have no doubt, from the character of the committee, that the work will be well done, and that the Congress will prove a pronounced success.

The State Medical Examining Boards, through their representatives, held a conference on Wednesday, May 6th, to devise ways and means for uniformity in the conduct of their duties. This meeting was presided over by Dr. J. H. Rauch, Secretary of the Illinois State Board of Health, who may justly be regarded as the father of the State Examining and Licensing Board System.

Dr. Picot, of North Carolina, was made Secretary of the conference, and an interesting session was held, at which representatives of the several States made many valuable suggestions as the result of their experience. It may be expected much good will come from the conference.

The Medical Editors held an interesting meeting, after which a dinner was enjoyed at Chamberlin's, on Monday night, May 4th. Dr. Frank Woodbury, of Philadelphia, was elected President for the ensuing year.

The social features of the meeting were necessarily of a high order, and embraced a reception by the physicians of Washington, on Tuesday night, at the Arlington, and one by Dr. Wm. A. Hammond at his elegant suburban villa, Belcourt, on Wednesday evening. The

Corcoran Art Gallery, the National Museum, and Mr. Thomas E. Waggaman's private collection were generously thrown open to the visitors. Surgeon-General Chas. Sutherland gave a reception at the Army Medical Museum, and an excursion was given to Mount Vernon. These, together with numerous private dinners and luncheons, afforded ample opportunity for social enjoyment. Many ladies were present, and fittingly graced these unofficial features of the meeting.

